# An unusual case of crusted scabies in an immunocompetent adult: A case report

**DOI:** 10.1002/ccr3.9325

**Published:** 2024-08-20

**Authors:** Martin Agyei, Priscilla Abrafi Opare‐Addo, Afua Ofori, Gloria Kyem, Solomon Gyabaah, Serwaa Asare‐Bediako

**Affiliations:** ^1^ Department of Internal Medicine Komfo Anokye Teaching Hospital Kumasi Ghana; ^2^ School of Medicine and Dentistry Kwame Nkrumah University of Science and Technology Kumasi Ghana

**Keywords:** Hyperparasitaemia, immunocompetent, LMICs, Norwegian scabies

## Abstract

**Key Clinical Message:**

Although rare, crusted scabies can affect immunocompetent individuals with no identifiable risk factors. A high index of suspicion, regardless of an individual's immunological status or absence of traditional risk factors, should be maintained by health professionals to facilitate prompt referral to a dermatologist.

**Abstract:**

Crusted scabies is an uncommon variant of human scabies characterized by extensive crusted and scaly hyperkeratotic papules, and plaques resulting from profound proliferation of mites in the skin. It is highly contagious and typically occurs in immunocompromised individuals. Reports of cases in healthy adults are rare. It is often resistant to routine treatment. We report a case of a 30‐year‐old immunocompetent male who presented to us with an extensive pruritic papular rash that started in the inter‐digital web spaces of his hands. Within 3 months from onset, the lesions progressed, and became hyperkeratotic, scaly, non‐pruritic, spreading to involve his entire body (sparing his face only). Crusty scabies was eventually diagnosed and treated by a dermatologist after months of misdiagnosis at peripheral facilities. Although rare, crusted scabies can affect immunocompetent individuals. A high index of suspicion is required, regardless of immunological status. Among immunocompetent individuals, a thorough search for traditional risk factors is imperative.

## Graphical abstract

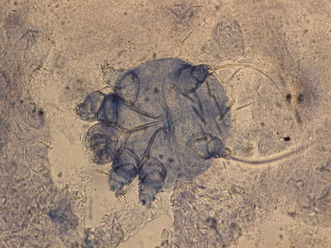

## 1 BACKGROUND

Human scabies is a skin infestation caused by an obligate human ecto‐parasitic mite; *Sarcoptes scabiei* var hominis, an arthropod in the class Arachnida, subclass Acari, family Sarcoptidae.^1^, ^2^ Crusted scabies is a highly contagious and intense variant, also known as Norwegian or Hyperkeratotic scabies. It differs from classic scabies in terms of appearance and severity.^3^ It is characterized by extensive crusted, hyperkeratotic papules, plaques, and nodules which results from hyperproliferation of mites. Compared to classical scabies, which is intensely pruritic, pruritus is typically minimal or absent in crusted scabies.^4^

Human scabies is caused by the transmission of the fertilized female scabies mite through close skin‐to‐skin contact for a few minutes and occasionally through fomites. The life cycle of the mite lasts for approximately 14 days.^5^ Female mites burrow into the stratum corneum of the skin to deposit feces and eggs. The eggs subsequently hatch into male and female nymphs which then mature and mate. While the males die almost immediately after mating, the females survive for about 2 months producing two or three eggs per day.^6^ Typically, only 15–20 mites are found on the skin of affected individuals due to scratching which physically removes the mites. Also, in the immune‐competent, the host *T* cell‐mediated immunity helps to prevent profound proliferation.^2^

The presence of certain risk factors that compromise host immunity and scratching capacity, leads to aggressive proliferation of mites, with numbers being as high as 1 million. These risk factors include immune suppressive states including human immunodeficiency virus (HIV) infection, graft‐versus‐host disease, malignancies, diabetes mellitus, immunosuppressive treatment, for example, corticosteroids, calcineurin inhibitors, and cytotoxic drugs may result in crusted scabies. Other predisposing factors include malnutrition, physical debilitation, advanced dementia, sensory or motor neuropathy, and mental retardation which lead to hyper‐parasitisation.^1^, ^3^, ^7^, ^8^

## 2 CASE REPORT

### 2.1 Patient information

A 30‐year‐old male trader presented to our dermatology clinic at the Komfo Anokye Teaching Hospital with a 3‐month history of generalized scaly skin eruption. He had initially noticed some pruritic papules in the webs of his fingers 3 months prior. These lesions rapidly spread over the period to involve the rest of his body sparing his face only. The Lesions eventually became hyperkeratotic and scaly. He had no history of chronic illness or contact with a person having a similar presentation. Drug history was unremarkable. This was his first episode of such a presentation.

Prior to being referred to our clinic, he had been seen at several peripheral health facilities where he was managed for toxic epidermal necrolysis, bullous pemphigoid, and ‘scaly dermatitis’ with no therapeutic success.

### 2.2 Clinical findings

**FIGURE 1:**
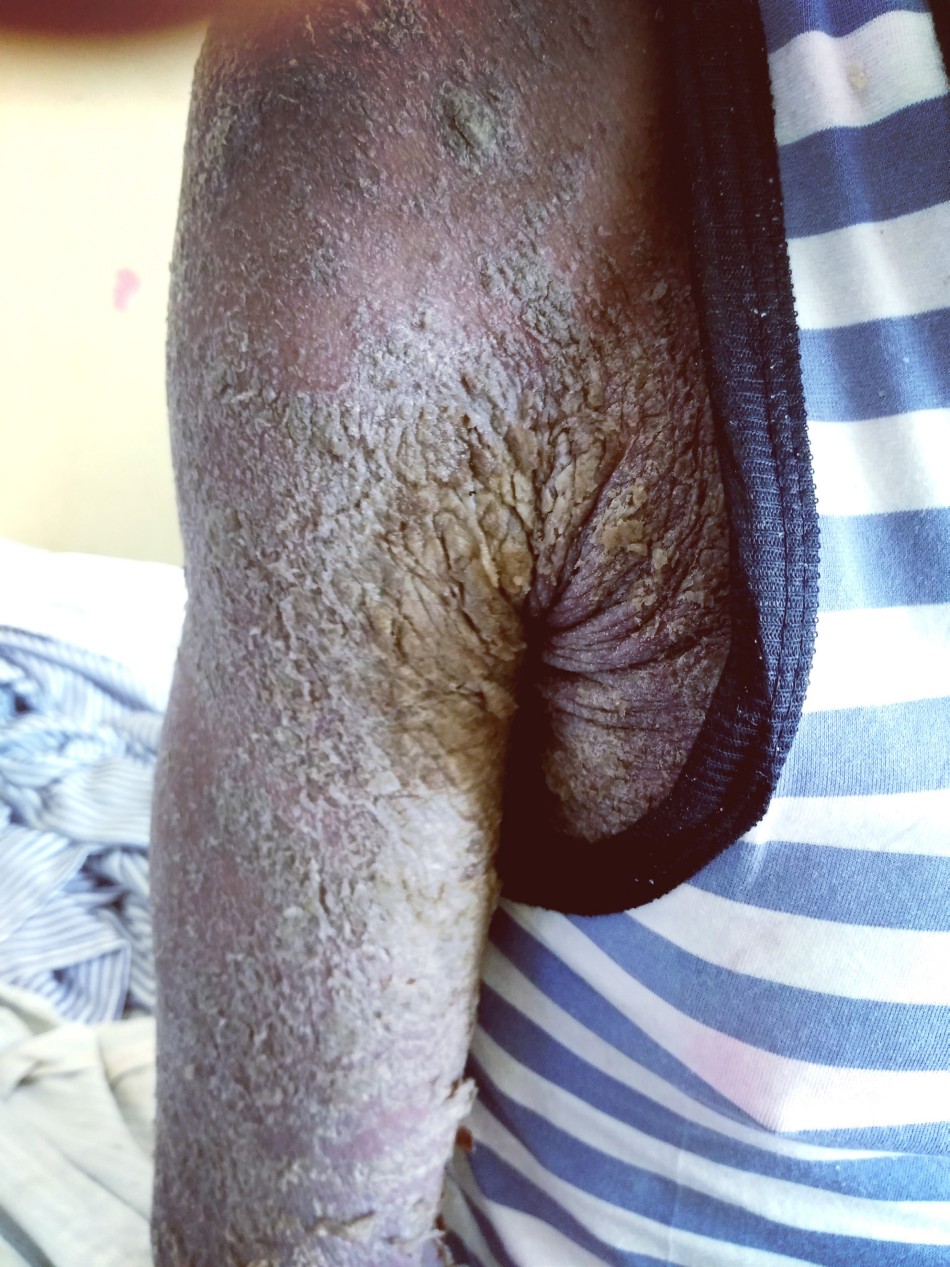
Crusted scabies involving the right upper limb.

**FIGURE 2:**
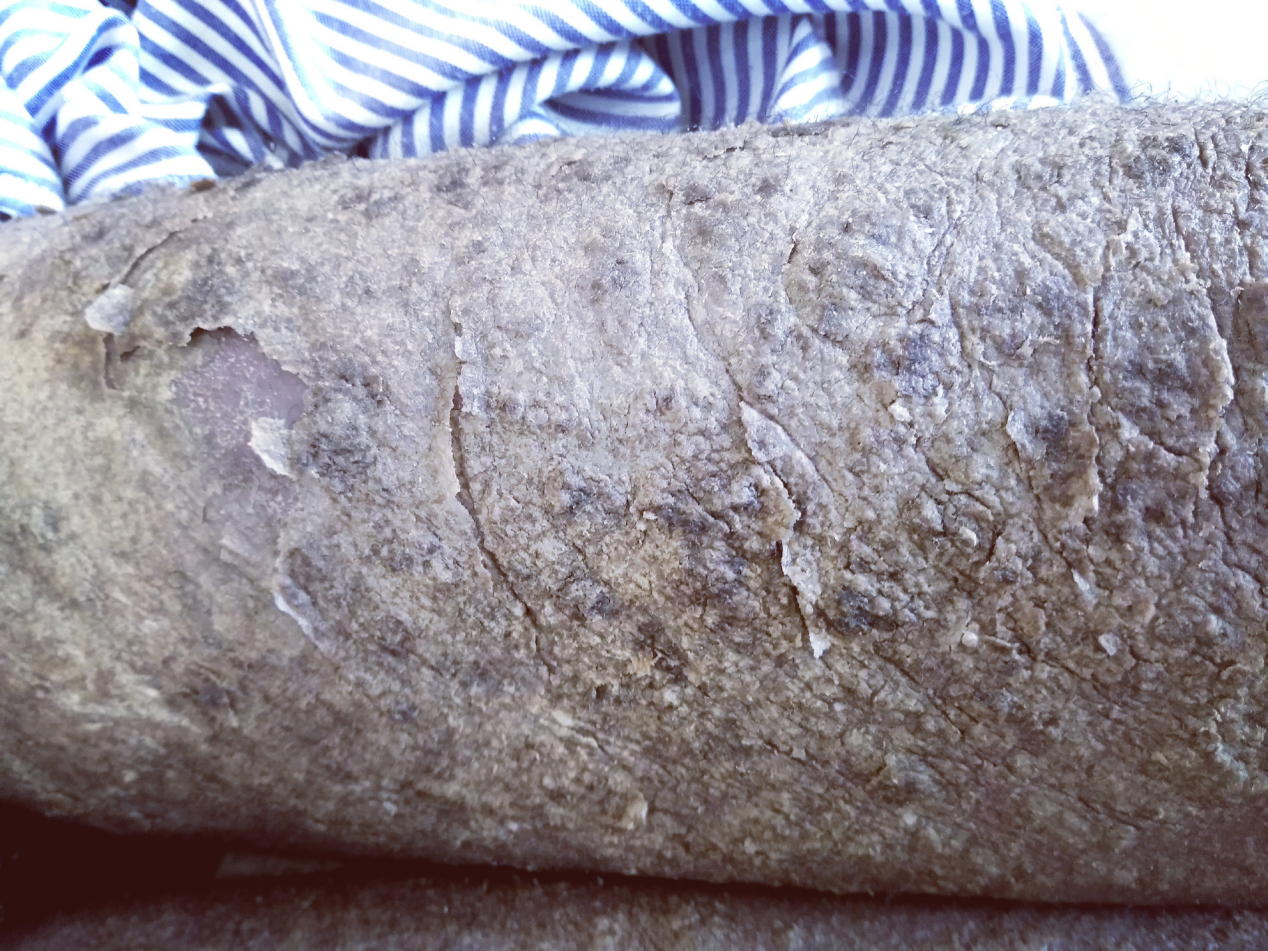
Crusted scabies involving the right lower limb.

On examination, extensive generalized sheet‐like scales with fissures were noticed all over his body, giving him the appearance of ‘a tree shedding its bark’ (Figures 1 and 2).

He was fully conscious with a temperature of 37.5°C, blood pressure of 124/81 mmHg, pulse of 118 beats per minute, and respiratory rate of 18 cycles per minute. Even though we could not objectively measure the patient's BMI, he appeared underweight.

### 2.3 Diagnostic assessment

**FIGURE 3:**
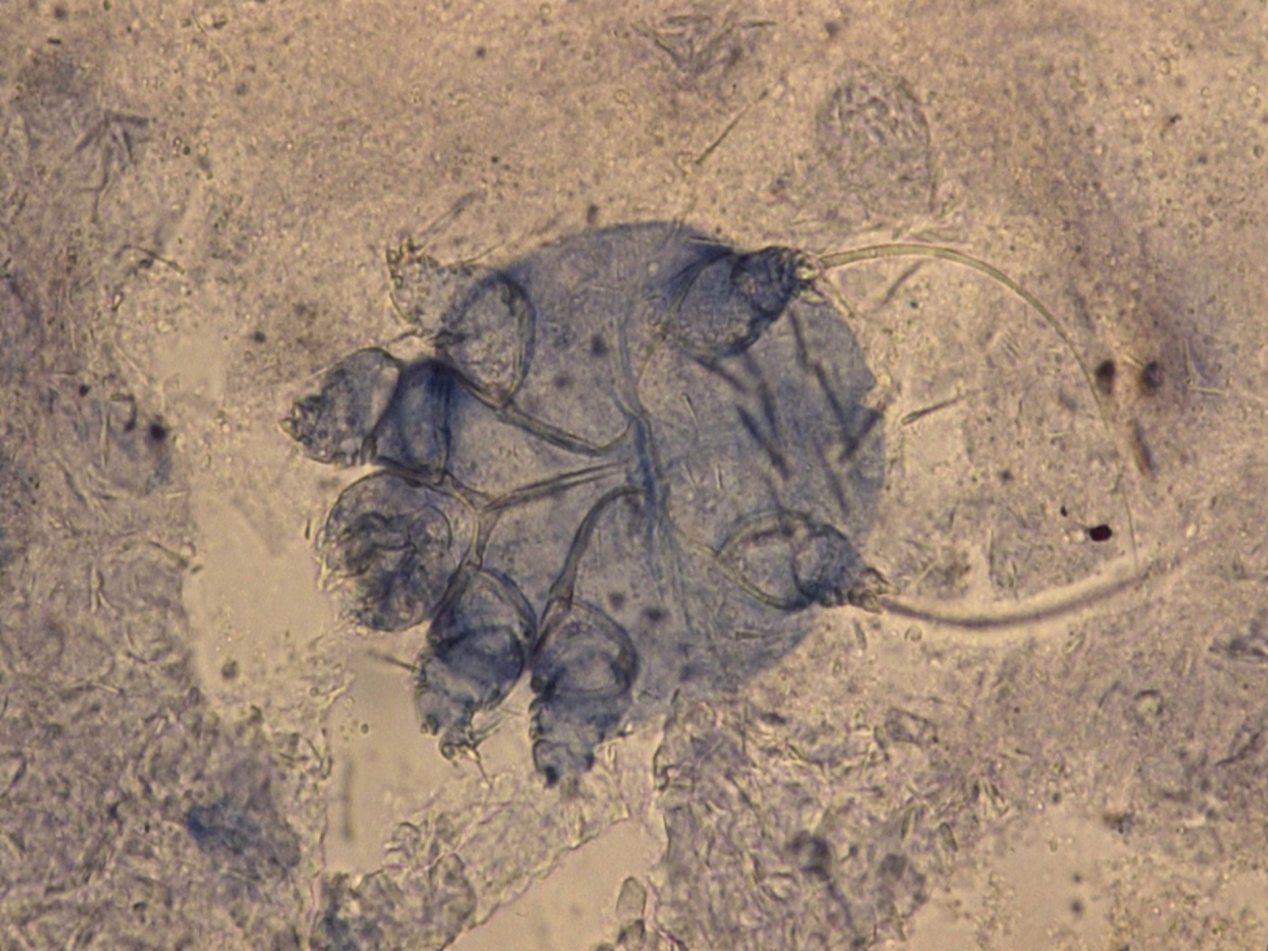
Wet preparation of skin scrapping showing scabies mite.

Wet preparation of skin scrapping for scabies mites showed numerous eggs and scabies mites. (Figure 3). Complete blood count revealed a white blood cell count of 9.32 × 10^3/uL with neutrophil and lymphocyte counts of 6.25 × 10^3/uL and 1.44 × 10^3/uL, respectively. Monocyte percentage was also elevated at 16.4%. Platelet count was 253 × 10^3/uL and hemoglobin concentration was 11.1 g/dL. All other parameters were within the normal range. HbA1c was 5.2%.

Antibody tests for HIV 1 and 2 (first response) done on two occasions were all negative. This was confirmed with PCR for HIV 1 RNA detection using the COBAS Ampliplep/Cobas TaqMan, which also yielded a negative result. PCR for the detection of HIV 2 and HTLV were unavailable. Serological test for Hepatitis B and C antibodies was negative. CD4, CD3, and CD8 counts were normal at 654, 924, and 270cells/mm^3^, respectively.

### 2.4 Therapeutic intervention

He received a stat dose of 12 mg oral ivermectin followed by four 12 mg doses for 4 weeks. KMnO_4_ baths and topical malathion (Derbac M) were also applied daily.

### 2.5 Follow‐up and outcome

The patient was discharged on the 6th day of admission due to the lack of a proper isolation unit. The social welfare team assisted him return home after his relatives abandoned him while on admission.

### 2.6 Limitations

Even though the patient showed significant improvement on admission, he never returned for his follow‐up visit.

## 3 DISCUSSION

Crusted scabies is a neglected tropical disease of global concern, frequently associated with misdiagnosis, lack of effective treatments, and growing resistance to standard treatments.^3^, ^9^

Even though crusted scabies usually begins from the inter‐digital web spaces of the fingers, unlike classical scabies, it progresses to involve the whole body sparing only the face as was the case in our patient.^10^ Lesions become crusted with subsequent flaking of layers of hyperparasitised skin, hence often misdiagnosed as eczema or psoriasis.^11^, ^12^ Our case was initially misdiagnosed as toxic epidermal necrolysis, bullous pemphigoid, and ‘scaly dermatitis’ prior to his referral to the dermatology clinic. This is further compounded by the fact that crusted scabies is typically non‐pruritic, unlike classical scabies. Its diagnosis therefore tends to elude most medical personnel. Various theories have been proposed to explain this; the most common being the loss of histamine‐mediated hypersensitivity to the mites and their products.^9^ A high index of suspicion is therefore required to initiate further evaluation for crusted scabies. Swift referral to the dermatologist is recommended in cases of uncertain diagnosis or treatment failure. This is especially important because prompt diagnosis helps to provide timely treatment to avoid complications, such as bacterial superinfection (generally by Streptococcus pyogenes) which can trigger sepsis, streptococcal glomerulonephritis, and/or rheumatic fever.^5^

Crusted scabies tends to occur in the presence of risk factors such as immunosuppression, malignancies, and diabetes mellitus.^1^, ^3^, ^7^, ^8^ Our patient was screened for diabetes, HIV, Hep B, and C which were all negative. HTLV could not be done because of resource constraints. CD4/CD8 were however within normal range. A plausible explanation is that the patient could have other unidentified risk factors such as the use of topical steroid‐containing medications. Even though the patient did not have any documented use of topical steroids such as triple action cream from the referring hospitals, it remains a possibility because it is the typical practice. When in doubt, general practitioners should refer patients to a dermatologist for additional evaluation rather than prescribing topical steroids when uncertain.

While Scabies infestation has no gender or racial predilection, it may be associated with other risk factors such as besides immunosuppression such as poor living conditions, overcrowded settlements, nursing homes, prisons, malnutrition, physical debilitation, and homelessness.^4^ Our patient had a history of occasional alcohol but there was no history of recreational drug use or psychiatric comorbidities. With regards to his occupation, he was a trader who retailed his stocks locally and did not frequently travel or lodge in hotels. We could not objectively measure the BMI however the patient appeared underweight and hence malnutrition could be a factor.

Crusted scabies may mimic several conditions including psoriasis, eczema and seborrhoeic dermatitis. While all these lesions are hyperkeratotic, they have some distinguishing features. The lesions in eczema are flexural in distribution and demonstrate lack on filaggrin on histopathological examination. Psoriatic lesions are scaly and found on the scalp, elbows, knees, and lower back, and typically associated with nail changes. Munro micro abscesses are a cardinal sign on histopathologic exam while Eggs, fecal pellets are present in crusted scabies. Our patient was initially misdiagnosed as toxic epidermal necrolysis, bullous pemphigoid, and ‘scaly dermatitis.’ Therefore it is essential to perform diagnostic tests such skin biopsy, dermoscopy, and direct microscopic visualization by a scraping technique also called Muller's test to confirm the diagnosis of crusted scabies after clinical diagnosis. Our choice of direct microscopic visualization was based on its high sensitivity and specificity, low level of complexity, and affordability.^13^

For the treatment of crusted scabies, a combination of systemic and topical agents (such as 5% permethrin cream and 25% benzyl benzoate) is recommended. Ivermectin is an oral antiparasitic agent approved for the treatment of worm infestations. Evidence suggests that oral ivermectin may be a safe and effective treatment for crusted scabies. Oral ivermectin (200 μg/kg/dose) should be taken with food. Depending on the severity of the infestation, ivermectin can be taken in three to seven doses. Permethrin, a synthetic pyrethroid that kills scabies mites and eggs, is the drug of choice for the treatment of scabies. Topical permethrin should be administered every 2–3 days for 1–2 weeks to treat crusted scabies. Benzyl benzoate may be used as an alternative topical agent to permethrin. However, this agent may cause immediate skin irritation. Keratolytic cream or agents may also be used to help reduce the crusting of the skin and aid in the absorption of topical permethrin or benzyl benzoate.^14^ They also aid in the prevention of superimposed bacterial infection. There is growing concern about the development of resistance to the currently available drugs.

Crusted scabies is extremely contagious, as the parasite load may be as high as 400 mites per gram of skin.^3^ Hence patient isolation, contact tracing and, treatment of asymptomatic close contacts with the index case is crucial to preventing institutional epidemic, spread among health workers, and other patients. The patient's home should be fumigated or kept locked for at least 4 days as the scabies mite cannot survive outside the human body beyond this period. Furred household pets such as dogs and cats may occasionally be infested but this self‐aborts in 2 weeks.^15^, ^16^ Our patient was appropriately diagnosed and treated 3 months after his first scabies symptoms appeared. By this time, he had visited more than four hospitals, graciously spreading the mites with each skin flake. Providing ideal isolation conditions for our patient was challenging because of resource constraints. Tracing of close contacts was also a challenge due to stigma and cultural barriers. Our patient's family members abandoned him the very day they brought him to the hospital believing he was under a curse. This highlights the need for public education.

## 4 CONCLUSION

Crusted scabies can affect immune competent persons. A high index of suspicion, regardless of an individual's immune status should be maintained by health professionals to facilitate prompt referral to a dermatologist. In such instances, a thorough evaluation for other risk factors is expedient. Public education is imperative to reduce the impact of stigma experienced by affected individuals.

## AUTHOR CONTRIBUTIONS

**Martin Agyei:** Conceptualization; data curation; investigation; methodology; project administration; supervision; validation; writing – original draft; writing – review and editing. **Priscilla Abrafi Opare‐Addo:** Conceptualization; data curation; methodology; project administration; supervision; validation; writing – original draft; writing – review and editing. **Afua Ofori:** Data curation; validation; writing – original draft; writing – review and editing. **Gloria Kyem:** Data curation; validation; writing – original draft. **Solomon Gyabaah:** Data curation; validation; writing – original draft. **Serwaa Asare‐Bediako:** Data curation; methodology; validation; writing – original draft; writing – review and editing.

## FUNDING INFORMATION

This research received no specific grant from any funding agency in the public, commercial, or not‐for‐profit sectors.

## CONFLICT OF INTEREST STATEMENT

The authors declare no conflict of interest.

## ETHICS APPROVAL

Ethical approval was obtained from the Kwame Nkrumah University of Science and Technology (KNUST) Committee on Human Research Publication and Ethics (CHRPE).

## CONSENT

Written informed consent was obtained from the patient to publish this report in accordance with the journal's patient consent policy.

## Data Availability

Data sharing is not applicable to this article as no datasets were generated or analysed during the current study.
